# Implementation of hospital governing boards: views from the field

**DOI:** 10.1186/1472-6963-14-178

**Published:** 2014-04-17

**Authors:** Zahirah McNatt, Jennifer W Thompson, Abraham Mengistu, Dawit Tatek, Erika Linnander, Leulseged Ageze, Ruth Lawson, Negalign Berhanu, Elizabeth H Bradley

**Affiliations:** 1 Yale University School of Public Health, 60 College Street, P.O. Box 208034, New Haven, Connecticut 06520-8034, USA; 2 Ethiopian Federal Ministry of Health, Addis Ababa, Ethiopia; 3 Abt Associates, Addis Ababa, Ethiopia; 4 Department for International Development, Abuja, Nigeria; 5 Jimma University College of Public Health and Medical Sciences, Jimma, Ethiopia

**Keywords:** Governance, Decentralization, Ethiopia, Healthcare reform

## Abstract

**Background:**

Decentralization through the establishment of hospital governing boards has been touted as an effective way to improve the quality and efficiency of hospitals in low-income countries. Although several studies have examined the process of decentralization, few have quantitatively assessed the implementation of hospital governing boards and their impact on hospital performance. Therefore, we sought to describe the functioning of governing boards and to determine the association between governing board functioning and hospital performance.

**Methods:**

We conducted a cross-sectional study with governing board chairpersons to assess board (1) structure, (2) roles and responsibilities and (3) training and orientation practices. Using bivariate analysis and multivariable regression, we examined the association between governing board functioning and hospital performance. Hospital performance indicators: 1) percent of hospital management standards met, measured with the Ethiopian Hospital Reform Implementation Guidelines and 2) patient experience, measured with the Inpatient and Outpatient Assessment of Healthcare surveys.

**Results:**

A total of 92 boards responded to the survey (96% response rate). The average percentage of EHRIG standards met was 58.1% (standard deviation (SD) 21.7 percentage points), and the mean overall patient experience score was 7.2 (SD 2.2). Hospitals with greater hospital management standards met had governing boards that paid members, reviewed performance in several domains quarterly or more frequently, developed new revenue sources, determined services to be outsourced, reviewed patient complaints, and had members with knowledge in business and financial management (all P-values < 0.05). Hospitals with more positive patient experience had governing boards that developed new revenue sources, determined services to be outsourced, and reviewed patient complaints (all P-values < 0.05).

**Conclusions:**

These cross-sectional data suggest that strengthening governing boards to perform essential responsibilities may result in improved hospital performance.

## Background

Decentralization has been touted as an effective means to improve the quality and efficiency of health systems in low-income countries [1,2]. However, evidence suggests that such efforts have failed due to a lack of clear governance roles and relationships [3]. In the context of hospitals, governing boards have been recommended as mechanisms by which strategic planning, financial management, and human resource management can be devolved from central authorities to local communities and provider organizations [4]. With adequate preparation and support, as well as meaningful community representation, hospital governing boards have been suggested as a means to increase community ownership, improve revenue generation, decrease expenses, and improve quality of care [5,6].

Although hospital governing boards have been widely discussed as part of healthcare reform in several low-income countries [7-10], we could find no peer reviewed studies on their implementation or impact in these settings. Nevertheless, reports from the gray literature have described the implementation of hospital governing boards in Lebanon, India, Indonesia, Zimbabwe, Kenya, and Ghana [7-9]. All reports highlighted the complexity of governing board implementation, but none provided quantitative data about their functioning or reported statistical associations between hospital governing board functioning and hospital performance.

Accordingly, we sought to describe the implementation of hospital governing boards in the context of national healthcare reform in a large, low-income country, Ethiopia. Ethiopia provided an ideal setting to explore topics related to decentralization to hospital governing boards because the Federal Ministry of Health (FMOH) and the Regional Health Bureaus (RHBs) established governing boards for the majority of government hospitals in 2005–2006. The Ethiopian government has been reforming the health sector for more than fifteen years, as guided by the Health Sector Development Program (HSDP). The nation faced many challenges in this endeavor including poorly funded facilities, shortages of clinical staff and limited access to services for rural communities. The vast majority of hospitals in Ethiopia have been funded and managed by the state, either through the federal government or regional health bureaus. The government has now focused on the decentralization of decision making from the federal level to the regional, zonal, and district levels, allowing for efficient mobilization of resources. A major element of this decentralization reform has been the establishment of governing boards, which reflect hospital and community leadership and are charged with strategic planning, budget approval, and performance management responsibilities.

We hypothesized that hospitals with higher functioning governing boards would have better performance, as measured by better adherence to the Ethiopian Hospital Reform Implementation Guidelines (EHRIG) and more positive patient experience. This is based on the concept that governing boards are local entities that can hold hospitals accountable for performance, can support strategic thinking to promote alignment between services and community needs, and can help advocate with the ministry for financial resources needed for hospitals. Despite substantial attention on the implementation of hospital governing boards in Ethiopia, previous studies have not examined how their actions may be linked empirically to hospital performance. Findings from this study can be used by policy makers and healthcare managers seeking to implement decentralization reforms in low-income countries.

## Methods

### Ethics statement

All research procedures were approved by the Institutional Review Board of the Yale School of Medicine (HIC Protocol #1105008523) and the Ethiopian Federal Ministry of Health.

### Setting

Nearly all government hospitals in Ethiopia have created governing boards, giving oversight and accountability responsibilities to community members, hospital managers, and other local government representatives. Hospital governing boards have 5–7 members, are required to consider gender and community representation in recruitment of new members, and exist to better mobilize resources, enhance community participation, and improve hospital performance. Board members are selected by the Federal Ministry of Health and Regional Health Bureaus as appropriate, serve 3–5 year terms, and focus on financial and operational oversight of hospitals, as per the Ethiopian Hospital Reform Implementation Guidelines [11] and legislation in 9 regional health bureaus and 2 city administrations. Several non-governmental organizations have supported the rollout of governing boards, including the orientation of boards to their roles and responsibilities. Only 16 of the government hospitals, all in smaller, more rural regions of Ethiopia, had not established governing boards at the time of the study; all hospitals with governing boards were contacted for the survey.

### Study design and sample

We conducted a cross-sectional study using quantitative data from a national survey of government hospital governing boards in Ethiopia during 2011 linked with hospital performance data from the Ethiopian national performance management system. The sampling frame included all government hospitals with governing boards (N = 100 of the 116 government hospitals in Ethiopia in 2011). We surveyed the chairperson or designee of the governing board in each hospital. Data were collected using face-to-face interviews and in rare cases telephone interviews (when distance or time posed a challenge, N = 3). A total of 92 hospital boards responded to the survey (response rate 96%). We also collected data (N = 85 hospitals) on hospital adherence to the EHRIG, 124 operational standards used to assess the management of government hospitals [11,12]. Additionally, for a subset of hospitals (N = 49), we collected data on patient experience, using a method and instrument previously validated for use in Ethiopia [13].

### Data collection and measures

#### Survey instrument

The study team developed a 36-question survey instrument to ascertain the functioning of Ethiopian hospital governing boards. Closed-ended survey items were developed through a rigorous, 6-month process, which included in-depth analysis of literature, convening of stakeholders, and review of policy documents in which intended functions of the governing boards were described. In addition, we included 1 open-ended item at the end of the survey, “Please share any other comments or concerns you have in relation to the functioning of your governing board.” In practice, the discussions focused on challenges and concerns.

The study team was multidisciplinary and consisted of academicians from universities in Ethiopia and the US, non-governmental organizations with knowledge in governance and hospital operations, and physicians, nurses, and individuals with hospital management experience in Ethiopia. Twelve interviewers traveled throughout Ethiopia, each independently conducting 1-hour face-to-face interviews with board Chairpersons or their designees. Challenges faced in interviewing were scheduling meetings with senior government officials and completing all survey questions when respondents were unsure of the correct response. To address these challenges, interviewers were persistent with follow up to officials and left responses blank if respondents did not know the answer. The survey also offered “I don’t know” as an optional response as appropriate.

The survey instrument was translated from English to Amharic by one individual, and a group of native Amharic speakers discussed the translation and made modifications. A separate individual back translated the Amharic version into English to ensure the translation was accurate. We performed cognitive interviews [14] in Amharic with five governing board members to ensure that the survey was comprehensive and comprehensible. These 5 board members were not included in the actual study. After the interviews, the survey was modified and interviewers underwent a 5 hour training session to prepare for data collection and potential interview challenges. The interview data were collected during a 1 month period between August and September of 2011.

#### Measures of governing board functioning

The survey included 3 domains of governing board functioning: (1) board structure, (2) board roles and responsibilities and (3) board training and orientation practices. The board structure domain included questions about the number of members, level of gender and community representation, members’ professional experience, meeting frequency, length, content and compensation. The board roles and responsibilities domain required respondents to describe what concrete actions were taken, with a focus on the review of specific clinical services and the monitoring of overall performance and what documents were created to outline roles and responsibilities (bylaws, terms of reference). The board training and orientation domain asked about the presence of orientation manuals and programs for new members, board self evaluation and additional training needed by board members.

#### Measures of hospital performance and hospital characteristics

We measured our outcome, hospital performance, using 2 indicators: (1) the percent of EHRIG standards met and (2) patient experience. The EHRIG includes 124 hospital management standards that assess 13 functions including leadership and governance, patient flow, medical records management, pharmacy services, laboratory services, nursing care, infection prevention, facilities management, medical equipment management, financial and asset management, human resource management, quality management and monitoring and reporting. The percent of EHRIG standards met has been collected and reported quarterly by government hospitals in Ethiopia since September 2010, with a subset being audited by RHBs.

Patient experience is routinely measured by the Federal Ministry of Health using validated surveys, which have been previously described [13]; hospital-level data were available for this study. The inpatient and outpatient surveys measured items in five domains including nursing communication, physician communication, physical environment, pain management and medication and symptom communication. Most questions were scored on a 4-point Likert scale, ranging from 1 (strongly disagree) to 4 (strongly agree). The item on overall satisfaction was measured on a scale of 0–10. Because the survey was relatively new, some hospitals experienced difficulty in reporting; thus these data were not available for 43 hospitals. Data for both indicators were collected from the FMOH through the national hospital performance monitoring and improvement system. All survey data were double-entered using a Microsoft Word template and imported into Microsoft Excel to examine and resolve discrepancies. We also measured several hospital characteristics: time since the implementation of the governing board, region in which the hospital was located, number of hospital beds, and type of hospital (primary, general and referral/tertiary).

#### Open-ended responses

In addition to the closed-ended responses to items pertaining to governing board functioning, we also asked an open-ended question, “Please share any other comments or concerns you have in relation to the functioning of your governing board.” This item was meant to probe for other areas in which the respondent thought the governing boards might need additional capacity built or not be functioning as well as possible.

#### Data analysis

We described the hospital sample using standard descriptive statistics for the overall sample and for each region. Using t-tests and simple linear regression, we examined unadjusted associations between hospital performance and the measures of governing board functioning. Hospital performance data on 2 indicators (the percent of EHRIG standards met and patient experience) were measured at the hospital level as part of the Federal Ministry of Health national hospital monitoring and performance improvement system. No patient-level data were available. We estimated adjusted associations between hospital performance and governing board functioning using multiple linear regression, adjusted for time since the governing board had been implemented, region in which the hospital was located, number of hospital beds, and type of hospital. We removed independent variables that were not significant (P-value > 0.05) and that did not add to the fit of the model, and we presented a parsimonious model. Data were analyzed using SAS Version 9.2.

## Results

### Hospital characteristics

Of the 100 hospitals approached, (managed by 97 boards), 1 had eliminated its governing board, leaving a potential sample of 96 boards for interview. Of these, 92 agreed to be interviewed (96% response rate), and the remaining 4 did not respond despite several contacts. Of the 92 respondents, 79 (89%) were board chair persons, and 13 (14%) were designees. A total of 43% (N = 40) of governing boards identified the hospital as a primary hospital and 9 of the 11 regions and city administrations were represented (Table 1). The average number of beds per hospital was 98.4 (standard deviation (SD) 64.3). The average EHRIG score was 58.1% of the standards being met (SD 21.7 percentage points). The average overall patient experience score was 7.2 out of 10 (SD 2.2).

**Table 1:** Sample of Ethiopian hospitals (N = 92)

**Governing board characteristics**	**N (%)**
Hospital type	
Referral/tertiary	18 (19.6%)
General	34 (36.9%)
Primary	40 (43.5%)
Region	
Addis Ababa	4 (4.4%)
Amhara	17 (18.5%)
Oromia	36 (39.1%)
Smaller regions*	4 (4.4%)
SNNP	18 (19.6%)
Tigray	13 (14.1%)

^*^Includes the regions/city administrations of Benishangul-Gumuz, Gambella, Dire Dawa and Harari.

### Governing board functioning

Nearly 80% of hospital governing boards paid board members for participation. The payment ranged from 0 USD to 25 USD per meeting. The average number of governing board meetings per year ranged from 3 in SNNPR to 13 in Addis Ababa. The percent of governing boards that conducted annual evaluations of the Chief Executive Officer (CEO) ranged from 0% in the smaller regions (Benishangul-Gumuz, Gambella, Dire Dawa and Harari) to 100% in the capital city of Ethiopia, Addis Ababa. Substantial geographic variation was apparent in the activities undertaken by governing boards (Table 2).

**Table 2:** Activities of Ethiopian hospital governing boards

**Governing board activities**	**Addis**	**Amhara**	**Oromia**	**Smaller**	**SNNP**	**Tigray**	**National**
N	4	17	36	4	18	13	92
Board Structure							
# of meetings per year (Mean)	13	7	9	7	3	5	7
% of boards that pay members	100	94	89	100	89	0	79
Roles and Responsibilities of Board							
% of boards that review patient experience quarterly or more often	75	44	49	50	50	55	50
% of boards that review quality of care quarterly or more often	75	56	54	75	50	50	59
% of boards that review referral services quarterly or more often	100	50	50	50	39	45	49
% of boards that develop new revenue sources	75	81	77	75	50	58	70
% of boards that determine services to be outsourced	100	79	44	25	39	58	52
% of boards that review patient complaints	75	69	77	25	29	55	61
% of boards that conduct annual performance evaluations of CEOs	100	50	71	0	22	83	57
% of boards that approve annual plans	100	100	97	100	94	100	98
Training and Orientation							
% of boards-all members need training-business/financial management	25	47	46	50	44	67	47
% of boards that have orientation manuals	0	63	58	25	0	58	43

### Associations between governing board functioning and hospital performance

Several board activities were associated with higher adherence to management standards (EHRIG) (Table 3) in the multivariable linear regression model adjusted for hospital type. Hospitals with greater adherence to EHRIG standards had governing boards that paid their members, reviewed hospital performance in several domains quarterly or more regularly, developed new revenue sources, determined services to be outsourced, reviewed patient complaints and had members with knowledge in business and financial management (all P-values < 0.05). In the multivariable linear regression model, adjusted for hospital type, hospitals with more positive patient experience scores had governing boards that developed new revenue sources, determined services to be outsourced, and reviewed patient complaints (all P-values < 0.05) (Table 4). In both models, time since the governing board was implemented, region in which the hospital was located, and number of hospital beds were not significant and were therefore dropped from the final models presented. Hospital type was associated with performance (with primary compared with secondary and tertiary hospitals having worse performance on both outcomes).

**Table 3:** **Adjusted associations between governing board activities and** % **of hospital management standards met**

**Governing board activities**	**% Management standards met**	**P-value**^**1**^
Receives payment		
Yes	61	P < 0.01
No	45	
Reviews referral services		
Quarterly or more frequently	65	P < 0.01
Less frequently	52	
Reviews patient experiences		
Quarterly or more frequently	64	P < 0.01
Less frequently	52	
Reviews quality of care		
Quarterly or more frequently	63	P < 0.05
Less frequently	52	
Develops new revenue sources		
Yes	61	P < 0.05
No	49	
Determines which services should be outsourced		
Yes	64	P < 0.05
No	52	
Reviews reports on patient complaints		
Yes	63	P < 0.01
No	48	
Needs training on business and financial management		
None or some members need training	64	P < 0.05
All members need training	52	

^1^P-value calculated from linear regression; results remained significant after adjusting for hospital type.

**Table 4:** Adjusted associations between governing board activities and patient experience

**Governing board activities**	**Mean patient experience score (0–10)**	**P-value**^**1**^
Develops new revenue sources		
Yes	6.1	P < 0.05
No	3.8	
Determines which services should be outsourced		
Yes	6.6	P < 0.01
No	4.1	
Reviews reports on patient complaints		
Yes	6.3	P < 0.05
No	4.3	

^1^P-value calculated from linear regression; results remained significant after adjusting for hospital type.

### Themes from open-ended responses about concerns in governing board functioning

A total of 80 individuals (87% of respondents) provided responses to the open-ended question. Several areas of concern regarding the implementation and current functioning of hospital governing boards emerged from these open-ended responses. Recurrent themes included: 1) unclear authority of the governing boards, 2) inadequate commitment and limited incentives for members to meet as a governing board, 3) ineffective communication and collaboration between the governing board and the regional health bureau, 4) unmet training needs of governing board members, and 5) inadequate representation from community, district, and zonal levels (as opposed to regional level) on the governing board (See Table 5 for larger set of quotations).

**Table 5:** Quotes identifying concerns related to hospital governing board functioning (open-ended responses)

**Theme #1. Unclear authority of the Governing Board (GB)**	
**Ambiguity in authority between GB and hospital management**	
**•**	The GB doesn't have authority to make decision on incentives.
**•**	Decisions made by GB have not implemented.
**•**	Even though hospital employees have ethical problems, the GB cannot take action because its role has not been clearly stated.
**•**	The role of GB in hospital should be stated clearly.
**•**	It would be good if the role and responsibility of GB and hospital management had clear demarcation.
**•**	What has been decided by the GB has not fully implemented.
**•**	The role of GB in taking actions on hospital employees when problems arise within in the hospital is not clearly stated.
**•**	The GB do not have full authority for every activity that took place in the hospital and if problems arose in the hospital, [they] do not taken corrective actions.
**Ambiguity in authority between the GB and regional health bureau (RHB)**	
**•**	The GB has been ordered direct procurement of drugs but the regional regulations did not allow that.
**•**	The GB has limited authority to take corrective actions on employees. This is in the authority of civil service and health office.
**•**	There is no autonomy; decisions made by the GB have been violated by the regional health bureau.
**•**	The GB cannot participate in drug control and auditing because the role of GB in this regard is unclear, and this has resulted in drug wastage.
**•**	Sometimes the regional economic and development bureau interfere in budgeting, which was the GB’s responsibility.
**•**	It would be better if the CEO could report directly to the GB instead of regional health bureau.
**Theme #2. Inadequate commitment and limited incentives for members to meet as a GB**	
**•**	Even though it is good to have a GB, the GB members are busy with their actual work; they do not have enough time to work for the GB.
**•**	In our zone, one person chairs three hospitals, which is inconvenient for the chair because he does not have enough time to get to know all information about the hospitals.
**•**	Most of GB members were high government officials; hence they do not give enough time to the GB committee.
**•**	Most of the governing board members are high government officials [and] they don’t [dedicate] enough time to the governing board.
**•**	The GB has not been meeting regularly.
**•**	The GB has never met every month [as it was supposed to] based on the legislation.
**•**	We have a shortage of time to monitor the hospital for we were busy.
**•**	Community representatives have not attended meeting as needed because they have private businesses.
**•**	The GB chair and members have been changed frequently.
**•**	We have concerns that some GB members may not be able to attend board meetings.
**•**	Inadequate payment for GB members.
**•**	Small GB members’ payment.
**•**	Little incentive payment to GB members.
**•**	There have been problems that the GBs were not performing well due to lack of payment
**Theme #3. Ineffective communication and collaboration between the GB and the RHB**	
**•**	The GB and RHB have to meet at least twice a year (inferring they do not).
**•**	It would be good if the GB authority was limited and had been controlled by the RHB.
**•**	[There is] no relationship between GB and the RHB; hospital data have exclusively been reported to the RHB (rather than to the GB and then to the RHB.
**•**	The GB reports to the RHB, so the RHB [should] work closely with the GB, follow challenges of the GB, solve financial problems, and human resource.
**Theme #4. Unmet training needs for GB members**	
**•**	Training should be given to GB members before they start work as GB members.
**•**	Give adequate training to employees in order to help them provide the community with faster service.
**•**	If GB members have received training on project designing… effective management style…how to give incentive to hospital staffs and retain them.
**•**	If the GB could receive all of the above-mentioned trainings.
**•**	[The GB needs] more capacity building training.
**•**	We lack of knowledge on how to lead hospital services because the GB members are not trained.
**•**	There should be orientation program for new GB members.
**•**	Training giving time and place should be convenient to members.
**•**	There should be an orientation program for new governing board members” and all additional training should be provided “on site, [rather] than outside of the district (another respondent).
**Theme #5. Inadequate representation from community, district and zonal levels on GB**	
**•**	If GB composition comprised more community representatives, [that would be better].
**•**	It would be better if the GB composition comprised more community representatives.
**•**	The GB members of district hospital are assigned by zone and reported to zone that implies the board [is not from the community] and does not have direct relationship with region and has less power.
**•**	It would be better if the GB members were nominated from the district [more local] administration than from zonal administration.
**•**	It would be better if the GB members for district hospital had been nominated from the district administration.
**•**	The current GB comprises members from the same district administration, so it would be better if the composition could from different districts [that the hospital serves].
**•**	According to the legislation GB chair for zonal/general hospital should have been from zonal administration, but due to distance from the zonal town and [because] the zonal administrators not were unable to come for the GB meetings, the current GB is chaired by district administrator.

### Unclear authority of the governing boards

More than 15 respondents indicated that they were concerned about the unclear authority of the hospital governing board. Some articulated boundary problems with the hospital management (being unclear about what was under the authority of the hospital management versus the governing board) and other respondents highlighted the boundary problems with the regional health bureaus (confusion over what was within the jurisdiction of the governing board versus what was the responsibility of the regional health bureau). The ambiguity in authority was apparent in statements about decisions concerning financial incentives for the staff, handling ethical issues, drug procurement, corrective action for employees, CEO supervision, and overall budgeting for the hospital. Quotations that illustrate ambiguity between the governing board and hospital management and the ambiguity between the governing board and the RHB include the following.

It would be good if the role and responsibility of GB and hospital management had clear demarcation (from one respondent)….even though hospital employees have ethical problems, the governing board could not take action because its role has not been clearly stated (from a second respondent).

It would be better if the CEO could report directly to the governing board instead of the regional health bureau (from one respondent)…Sometimes the regional economy and development bureau interfere with budgeting, which was a board responsibility (from a second respondent)…The GB has no autonomy; decisions made by the governing board have been violated by the RHB (from another respondent).

### Inadequate commitment and limited incentives for governing board members

A second issue described by respondents was lack of commitment of board members to meet regularly and to attend meetings. Respondents suggested the incentives were insufficient. In some cases, chairs were overcommitted to more than one board; in other cases, members were high-level government officials from the zonal or regional levels, who were too busy to prioritize their governing board responsibilities. Community members were also described as sometimes too busy to commit to attending hospital board meetings, and respondents highlighted the lack of financial incentives for board members being a detriment to the functioning of the board. For instance, respondents stated the following:

In our zone, one person chairs three hospitals, which is inconvenient for the chair because he does not have enough time to get to know all information about the hospitals (one respondent)…Most of the governing board members are high government officials [and] they do not [dedicate] enough time to the governing board (a second respondent)… Community representatives have not attended meetings as needed because they have private businesses (another respondent)…[The GB is not performing well due to] inadequate payment (another respondent).

### Ineffective communication and collaboration with RHB

In addition to ambiguity about the governing board versus the RHB roles, lack of communication and collaboration between the entities was also described as limiting board functioning. Although this concern was less frequently noted, respondents expressed that the relationships could be more effective in some cases.

[There is] no relationship between GB and the RHB; hospital data have exclusively been reported to the RHB (rather than to the GB and then to the RHB (one respondent)…The GB reports to the RHB, so the RHB [should] work closely with the GB, follow challenges of the GB, solve financial and human resource problems (a second respondent).

### Unmet training needs of governing board members

A central concern for respondent was the lack of training and orientation for governing board members. Respondents believed that often board members did not have the needed background or training to be effective in the position. The following illustrative statements highlight this theme.

Training should be given to GB members before they start work as GB members (on respondent)…If GB members have received training on project designing… effective management style…how to give incentive to hospital staffs and retain them, [they would do a better job] (a second respondent)…There should be an orientation program for new governing board members and all additional training should be provided on site, [rather] than outside of the district (another respondent).

### Inadequate representation from community, district, and zonal levels

Several respondents described concerns about insufficient representation of the community or more local levels of government on the hospital governing board. The majority of respondents who identified this issue stated more community representation was needed, although some also thought greater diversity across districts was required, particularly for hospitals that served people from multiple districts. Overall, the sense of those concerned was that the governing board did not have adequate representation from the communities the hospital served and still depended too much on leadership from the regional levels of government. Respondents stated:

It would be better if the GB composition comprised of more community representatives (one respondent)…It would be better if the GB members were nominated from the district [more local] administration than from zonal administration (a second respondent)… The current GB comprises members from the same district administration, so it would be better if the composition could from different districts [that the hospital serves] (another respondent).

## Discussion

We found significant associations between 8 key board activities and hospital performance, as measured by adherence to management standards and more positive patient experience. In particular, our findings suggest that strengthening governing boards to perform essential financial and operational responsibilities may result in improved hospital performance. Additionally, the findings highlight some of the ongoing concerns that may be experienced by countries seeking to decentralize including needs for reinforcing clear distinctions between roles of regional government, governing boards, and hospital management and supporting board members with sufficient training and incentives to engage in governance activities. Our findings add to the body of knowledge about hospital governing boards in low-income settings by explicitly outlining implementable board activities that are associated with better hospital performance.

The board activities we found to be associated with hospital performance pertained broadly to two concepts, board attention to financial concerns and consistent board review of operational issues. These results may be due to the fact that boards with the authority and ability to make financial decisions may be better able to mobilize resources and meet the needs of staff and patients. Additionally, boards which frequently review hospital operations may identify problems and respond rapidly, resulting in more immediate solutions.

Our findings are consistent with literature about governing boards in high-income settings to the degree we find that board activities are associated with hospital performance. The literature in high-income settings suggests that effective boards, as measured by the Board Self Assessment Questionnaire (BSAQ), are able to lower expenses and increase profitability in their hospitals; furthermore, hospital boards with more corporate configurations are less likely to experience closure [5,15,16]. We found, however, that although governing boards in Ethiopia had limited ability to control financial decisions (in comparison to their counterparts in high income countries), they nonetheless had significant influence on hospital operations. Thus, our study demonstrates additional ways in which hospital governing boards in low-income settings may be important, beyond their financial responsibilities.

We additionally identified a set of challenges as perceived by governing board chairs responding to an open-ended question. These focused on role definition and ambiguity in authority of the governing board, which in some cases seemed to risk eclipsing the roles and authority of hospital management and in other cases were limited by inadequate authority over financial and human resource decisions still under the jurisdiction of regional health bureaus. These findings highlight the central challenge in decentralization efforts, which is coming to terms with the roles and responsibilities – both on paper and in practice – of new governance structures between the regional government and the communities that hospitals serve. In Ethiopia, the reform efforts have progressed substantially to create governing boards and, in so doing, improve hospital management and patient experience; however, qualitative data suggest that the issues of governing board member selection, training, and motivation, as well as clarity in responsibility persist in some cases and will require ongoing attention to reinforce the vision of decentralization.

Our findings should be interpreted in light of several limitations. First, although we achieved a high response rate (96%), our sample size was modest given the limited number of government hospitals in Ethiopia; this was even more challenging for the analysis of patient experience because only 49 of the hospitals reported. However, we did include in the analysis all hospitals that collected these data. Second, our study was cross sectional and therefore should not be interpreted as establishing causal relationships, although the findings set a foundation for future, longitudinal studies of hospital performance. Third, the survey data were self-report by governing board chairpersons or designees and could not be corroborated through direct observation of board proceedings or minutes. Our data should therefore be understood as documenting the respondents’ perceptions of governing boards’ functioning. Subsequent research may benefit from more comprehensive interviewing of all board members. Last, the open-ended data attained were helpful but we were unable to have in-depth qualitative interviews of both the successes and challenges encountered with establishing and managing governing boards. Such a study would provide added depth to the understanding of this largely quantitative analysis. Despite these limitations, this exploratory study is the first of which we know to document statistical associations between hospital governance reform and hospital performance in Africa.

## Conclusions

Strengthening hospital governing boards to perform essential activities could result in improved hospital performance, in a variety of areas including quality and finance. Our findings indicate that newly established boards with limited resources may be best served by focusing efforts on key board activities that are proven to influence hospital performance, rather than attempting to accomplish all governance responsibilities in the short term. Further analysis is required to better understand the spectrum of responsibilities that can be delegated to hospital governing boards and how the varying levels of delegation can influence hospital performance. Results of this study can inform policy makers seeking to implement strategies to support decentralization to the hospital level.

## Abbreviations

CEO: Chief Executive Officer; EHRIG: Ethiopian Hospital Reform Implementation Guideline; FMOH: Federal Ministry of Health; GB: Governing Board; RHB: Regional Health Bureau; IPAHC/OPAHC: Inpatient/Outpatient Assessment of Healthcare; BSAQ: Board Self Assessment Questionnaire.

## Competing interests

BB, AM and LA participated in the drafting of Ethiopian legislation that established hospital governing boards. AM served on a federal hospital governing board in Addis Ababa (federal hospitals were not included in the study).

## Authors’ contributions

ZM, EL, RL, and BB conceptualized the paper. ZM implemented the study under the supervision of BB. The data collection was led by ZM with support from various personnel listed in the acknowledgements. The data analysis was carried out by JWT. ZM drafted the manuscript with revisions by BB. EL, DT, RL, LA, NB and AM reviewed and edited the paper. All authors read and approved the final manuscript.

## Pre-publication history

The pre-publication history for this paper can be accessed here:

http://www.biomedcentral.com/1472-6963/14/178/prepub

## References

[B1] Bossert TJ Decentralization and governance in health. USAID Health 2020 Policy Brief 2008 http://www.healthsystems2020.org/content/resource/detail/1974

[B2] Partners for Health Reformplus Insights for Implementers: decentralization and health system reform, Issue in brief 2002 Bethesda, Maryland: Abt Associates

[B3] Ramesh M Wu X He AJ Health governance and healthcare reforms in China Health Policy Plan 2013 doi:10.1093/heapol/czs109 10.1093/heapol/czs109 23293100

[B4] Bossert TJ Beauvais JC Decentralization of health systems in Ghana, Zambia, Uganda and the Philippines: a comparative analysis of decision space Health Policy Plan 2002 14 1 14 31 11861583 10.1093/heapol/17.1.14

[B5] McDonagh KJ Hospital governing boards: a study of their effectiveness in relation to organizational performance J Healthc Manag 2006 14 6 377 389 17184002

[B6] Collins D Njeru G Meme J Newbrander W Hospital autonomy: the experience of Kenyatta National Hospital Int J Health Plann Manage 1999 14 2 129 153 10538935 10.1002/(SICI)1099-1751(199904/06)14:2<129::AID-HPM541>3.0.CO;2-R

[B7] Eid F Hospital governance and incentive design: the case of corporatized government hospitals in Lebanon. Policy Research Working Paper 2727 2001 The World Bank: Washington, D.C

[B8] Govindaraj R Obuobi A Enyimayew N Antwi P Ofsu-Amaah S Hospital autonomy in Ghana: the experience of Kore Bu and Komfo Anokye teaching hospitals. Data for Decision Making Project 1996 http://www.hsph.harvard.edu/ihsg/publications/pdf/No-41.PDF

[B9] Govindaraj R Chawla M Recent experiences with hospital autonomy in developing countries -- what can we learn? Data for Decision Making Project 1996 http://apin.harvard.edu/ihsg/publications/pdf/No-32-2.pdf

[B10] Sarp N An example of health sector reforms in Turkey: hoslital decentralization (health enterprises) J Anakara Med Sch 2002 14 1 9 18

[B11] Federal Democratic Republic of Ethiopia, Ministry of Health Ethiopian Hospital Management Initiative: Ethiopian Hospital Reform Implementation Guidelines 2012 14 2012

[B12] Federal Democratic Republic of Ethiopia, Ministry of Health Ethiopian Hospital Management Initiative: Ethiopian Hospital Reform Implementation Guidelines 2010 14

[B13] Webster TR Mantopoulos J Jackson E Cole-Lewis H Kidane L Kebede S Abebe Y Lawson R Bradley EH A brief questionnaire for assessing patient healthcare experiences in low-income settings Int J Qual Health Care 2011 14 3 258 268 21531989 10.1093/intqhc/mzr019

[B14] Krause N A comprehensive strategy for developing closed-ended survey items for use in studies of older adults J Gerontol B Psychol Sci Soc Sci 2002 14 5 S263 274 12198106 10.1093/geronb/57.5.s263 PMC3046550

[B15] Alexander JA Lee SY Wang V Margolin FS Changes in the monitoring and oversight practices of not-for-profit hospital governing boards 1989–2005: evidence from three national surveys Med Care Res Rev 2009 14 2 181 196 19052168 10.1177/1077558708326527

[B16] Alexander JA Ye Y Lee SY Weiner BJ The effects of governing board configuration on profound organizational change in hospitals J Health Soc Behav 2006 14 3 291 308 17066778 10.1177/002214650604700307

